# Association of genotypes with infection types and antifungal susceptibilities in Candida albicans as revealed by recent molecular typing strategies

**DOI:** 10.1080/21501203.2014.899525

**Published:** 2014-03-25

**Authors:** Feng-Yan Bai

**Affiliations:** State Key Laboratory of Mycology, Institute of Microbiology, Chinese Academy of Sciences, Beijing 100101, China

**Keywords:** *Candida albicans*, candidiasis, molecular typing, molecular epidemiology

## Abstract

*Candida albicans* is a commensal microorganism in the mucosa of healthy individuals, but is also the most common opportunistic fungal pathogen of humans. It causes from benign infections such as oral and vaginal candidiasis to fatal, systematic diseases in immunocompromised or critically ill patients. In addition to improved therapy, the rapid and accurate identification of the disease-causing strains is crucial for diagnosis, clinical treatment and epidemiological studies of candidiasis. A variety of methods for strain typing of *C. albicans* have been developed. The most commonly used methods with the focus on recently developed molecular typing or DNA-fingerprinting strategies and the recent findings in the association of specific and genetically similar genotypes with certain infection types and the correlation between azole susceptibilities and certain genotypes of *C. albicans* from China are reviewed.

## Introduction

*Candida albicans* is a dimorphic fungus which usually occurs as a commensal microorganism in the mucosa of humans. It can be found in the intestinal and urogenital tracts and the oral cavities of healthy individuals. The species is also the most common opportunistic fungal pathogen of humans. It causes from benign infections such as oral and vaginal candidiasis to fatal, systematic disease in immunocompromised or critically ill patients. Nearly three-quarters of all healthy women experience at least one episode of *Candida* vulvovaginitis during their lifetime and about 5–10% of women endure recurrent bouts of the disease (Sobel 2007). Due to the growing population over the last two to three decades of transiently or permanently immunocompromised patients, invasive infections caused by *C. albicans* have become an increasingly important clinical problem. It is recognized as the fourth leading cause of nosocomial infections (Pfaller et al. 1998; Edmond et al. 1999). As a result of the difficulties in the early accurate diagnosis of systemic candidiasis, a limited number of suitable and effective antifungal drugs and the increasing drug resistance of the etiologic agents, mortality rates from systemic candidiasis remain high (Gudlaugsson et al. 2003). Even mucosal infections caused by *C. albicans,* such as vaginal candidiasis, often constitute a management problem because of the high rate of recurrence. Fundamental issues with regard to diagnosis, epidemiology and pathogenesis of both mucosal and invasive candidiasis remain to be elucidated.

In addition to improved therapy, the rapid and accurate identification of the disease-causing strains is crucial for diagnosis, clinical treatment and epidemiological studies of candidiasis. A variety of methods for typing strains of *C. albicans* have been described. The most commonly used methods with the focus on recently developed molecular typing or DNA-fingerprinting strategies and the recent findings in the association of specific and genetically similar genotypes with certain infections and the correlation between azole susceptibilities and genotypes of *C. albicans* from China are discussed in this review.

## Biotying based on phenotypic characters

The methods based on phenotypic characteristics, referred to as biotyping methods, were first employed for strain typing of *C. albicans.* Serotyping based on antigenic properties is one of the first biotyping strategies used to discriminate among *C. albicans* strains. This strategy was used to type strains for a few decades and three serotyping methods were established for *C. albicans,* including Hasenclever's antisera HSN1 and HSN2 (Hasenclever et al. 1961a, 1961b), the Iatron *Candida* Check factor 6 typing antiserum (IF6) (Poulain et al. 1985) and agglutination with the monoclonal antibody H9 (Brawner & Cutler 1989). However, the discriminatory power of these methods is limited. Separation of an entire species into a few groups does not provide meaningful resolution for the majority of epidemiological questions to be addressed. In addition, it was found that antigen expression could be affected by phase of growth and culture conditions, which placed in question the serotyping methodology (Poulain et al. 1985; Brawner 1991).

Odds and Abbott, in the early 1980s, developed a complex biotyping protocol for distinguishing among *Candida* species and among strains of a species based on a set of physiological assays (Odds & Abbott 1980, 1983). The assays testing for growth at pH 1.4; production of secreted acid proteinase; resistance to flucytosine, boric acid and safranin; assimilation of urea, sorbose and citrate; and sensitivity to high salt were originally developed for discriminating among species. Four additional assays, including resistance to tetrazolium salts, sodium periodate and cetrimide and growth on MacConkey agar, were then used as supplementary tests for discrimination among strains within a *Candida* species. The protocol was modified by Childress et al. (1989) to make it more amenable to general use. This biotyping strategy was effectively used in a number of epidemiological studies (Odds et al. 1987, 1989b; Korting et al. 1988; Poirier et al. 1990; Xu & Samaranayake 1995; Lipperheide et al. 1996). However, the system was found to have poor interlaboratory reproducibility (Odds et al. 1989a); the use of the biotyping method in clinical research is therefore limited.

In addition to the serotyping and biotyping methods mentioned above, a number of other typing methods based on phenotypic characters have been used for *C. albicans* strain discrimination, namely morphotyping, sugar-assimilation typing, killer yeast typing, resistotyping and drug-susceptibility typing (see Soll 2000). Although theoretically the phenotype reflects genetic background of an organism, biotyping methods have fundamental problems that limit their use in epidemiological investigation. First, results of the tests, even the expression of serotypes, can be easily affected by growth conditions (Poulain et al. 1985; Brawner & Cutler 1989). If the growth conditions and time of cell harvesting are not precisely controlled and duplicated among experiments and laboratories, intralaboratory and interlaboratory reproducibility of the results obtained from biotyping methods will be compromised. Second, a number of general phenotypes of *C. albicans* that affect a variety of phenotypic traits undergo spontaneous high-frequency switching (Slutsky et al. 1985; Soll 1992), which may result in phenotypic differences for the same strain grown under the same conditions.

## Multilocus enzyme electrophoresis

Multilocus enzyme electrophoresis (MLEE) assesses isozyme or allozyme polymorphism by starch gel electrophoresis, polyacrylamide gel electrophoresis or isoelectric focusing under native conditions. The enzymes are visualized in the gels by specific enzyme-staining procedures. This method directly reflects allelic differences at defined loci. Any enzyme (protein) that can be selectively stained can be the target of analysis. If the protein bands are variable, they are considered to be alleles on the basis of mobility. Both alleles in a diploid can be observed; thus, the method can assess codominant markers in diploids for each locus. If enzymes are carefully selected, MLEE can discriminate among the gene products of different alleles for a number of loci. For instance, Pujol et al. (1997) tested 21 enzymes of *C. albicans* using MLEE on 29 isolates. Thirteen exhibited variability and were therefore used in the analysis. This study demonstrated further that if enough markers are used, MLEE will reveal microevolution within strains (Pujol et al. 1997). The technique has been effectively used to fingerprint *C. albicans* and a number of other *Candida* species (Taylor et al. 1999; Soll 2000). Although the enzyme patterns detected by MLEE are actually phenotypes, the protocol outperforms several popular DNA-fingerprinting methods in assessing genetic relatedness among strains.

The shortcomings of this method include: (1) it is relatively time-consuming, because at least 10 enzymes that provide variability among isolates must be analysed; (2) it assays the variability at protein level, so that much variation at the nucleotide level may go undetected because nucleotide substitutions do not necessarily change the amino-acid composition and (3) changes in amino-acid composition do not necessarily change the electrophoretic mobility of the protein and, as a consequence, alleles that are considered to be the same protein alleles from different individuals may represent different gene alleles.

## Electrophoretic karyotyping

The numbers of chromosomes in the cell as well as deviations from the basic number provide useful information for genetics and systematics of organisms. The use of the information in fungi was hampered because of the difficulty in counting the chromosomal number in fungal cells by using cytological methods as usually used in higher plants and animals. The invention of pulsed-field gel electrophoresis (PFGE) and other similar systems in the middle of 1980s partially resolved the problem (Schwartz & Cantor 1984). Chromosome-sized DNA fragments of the yeast genome can be readily separated according to size in a gel with the PFGE apparatus such as contour-clamped homogeneous electric field gel electrophoresis, and can be visualized by ethidium-bromide staining. The chromosomal numbers and genome sizes of many fungi have been estimated by using this method (Walz 1995). A striking discovery is that most fungal species have rather variable karyotypes among strains. The polymorphism has been observed in both asexual and sexual fungi and most likely results from both mitotic and meiotic processes. The phenomenon which is called chromosome length polymorphism has been employed for fingerprinting of yeast strains.

Electrophoretic karyotyping has been used extensively for *C. albicans* strain typing (see Soll 2000, for references). Sangeorzan et al. (1995) demonstrated that patterns generated by electrophoretic karyotyping were highly reproducible among experiments, relatively insensitive to preparation methods within the same laboratory, and unaffected by high-frequency phenotypic switching. Electrophoretic karyotyping has been shown to be the most powerful method for *Saccharomces cerevisiae* strain typing (Schuller et al. 2004); however, its discriminatory ability for *C. albicans* strains is limited because of the relatively lower chromosomal number and larger molecular sizes of individual chromosomes of the species. The protocol cannot distinguish chromosomal DNA molecules with slight differences in sizes. The discriminatory power of electrophoretic karyotyping as a fingerprinting method can be increased dramatically by digesting chromosome-length DNA with endonucleases prior to PFGE (Cormican et al. 1996; Pontieri et al. 1996). By increasing the complexity of the pattern in this way, electrophoretic karyotyping could serve as an effective fingerprinting system.

## Restriction fragment length polymorphism analysis

Restriction fragment length polymorphism (RFLP) is one of the first molecular methods used to assess genetic diversity or relatedness of strains within a pathogenic fungal species. RFLP analysis shows the DNA-sequence variation by electrophoretic-banding patterns of DNA samples which are digested by one or more restriction enzymes. Restriction enzymes recognize very specific sequences of nucleotides in DNA. DNAs from different individuals rarely have exactly the same array of restriction sites and distances between these sites. By cutting a DNA sample with a particular restriction enzyme, DNA fragments of different length are obtained. These fragments are separated by gel electrophoresis, resulting in a pattern of bands that is unique for the particular DNA being analysed. Any region of DNA (locus) can be used for RFLP analysis. Restriction patterns can be generated directly if the target exists in multiple copies, for example, mitochondrial DNA (mDNA) or nulear rDNA, or, more frequently, through two indirect approaches. First, the total nuclear DNA is subjected to gel electrophoresis after being digested with a particular restriction enzyme, transferred to a membrane by southern blotting and then hybridized with a radioisotope-labelled particular DNA probe; the banding pattern is finally visualized by radiography. The second approach called polymerase chain reaction (PCR)-RFLP is simpler: A specific gene or DNA fragment is amplified by PCR and then digested with particular restriction enzymes; the fragments are then separated by gel electrophoresis and visualized by ethidium-bromide staining.

RFLP analysis has been extensively applied to fingerprint strains and to assess population structure, mode of reproduction and microevolution of *C. albicans* and related species (Taylor et al. 1999; Soll 2000; Xu 2006). A variety of probes have been developed including singlegene probes, rDNA probes, mDNA probes and repetitive and complex DNA probes (Soll & Pujol 2003). DNA fingerprinting with the complex probe Ca3 has revealed five *C. albicans* clades with geographical specificity: clades I, II, III, SA and E. Clade SA is relatively specific to or highly enriched in South Africa; clade E is relatively specific to Europe; and clade II is absent in the Southwest USA and South America. The results of these studies also have identified clade-specific drug resistance. The majority of isolates within clade I are moderately or highly resistant to 5-fluorocytosine, while the great majority of isolates within the four other clades (II, III, SA, E) are not (Soll & Pujol 2003). These results showed the promising of RFLP analysis with complex DNA probes in assessing the population structure of *C. albicans* worldwide. However, the use of this method is limited by the complexity of analysis procedure, the need of radioisotopes and the difficulty in interlaboratory comparison. RFLP analysis without hybridization probes, especially PCR-RFLP is straightforward and easy to use without the requirement of special equipment. However, the discriminative power of the analysis is usually limited and sequence differences occurred outside the restriction sites cannot be detected, consequently, fragments with identical sizes do not necessarily have identical sequences.

## Randomly amplified polymorphic DNA

Randomly amplified polymorphic DNA (RAPD) or arbitrarily primed PCR (AP-PCR) analysis assays DNA sequence variation in PCR-priming regions. RAPD analysis uses one short PCR primer (ca. 10 bp) and a low annealing temperature to generate several fragments in one reaction. Nucleotide substitutions in the PCR-priming regions, particularly the 3′ ends, can prevent primer annealing and PCR amplification, resulting in the differences in PCR products. Amplicons are separated on an agarose gel and stained with ethidium bromide. RAPD is technically simple but its resolving power can be dramatically increased by increasing the number of oligonucleotide primers that provide variability among independent isolates. It often detects variation among isolates that are invariant with RFLP analysis, and thus it has evolved as the most popular method for DNA fingerprinting the infectious fungi, including *C. albicans* (for references see Taylor et al. 1999; Soll 2000; Xu 2006) and other *Candida* species. However, a number of shortcomings of RAPD analysis must be considered.

The main drawback of RAPD analysis is poor reproducibility. The problem may occur not only among laboratories but also within a laboratory over time. Almost every technical aspect of PCR can affect reproducibility of RAPD analysis. Another problem is that bands of equal electrophoretic mobility may not be homologous. Rieseberg (1996) demonstrated that a disturbingly large fraction (13%) of RAPD bands with equal mobility from hybrid plants were not homologous. Sequencing of the RAPD bands has been used to confirm their identity (Burt et al. 1996; Graser et al. 1996).

## Microsatellite polymorphisms

In recent years, short tandem repeats or microsatellites have been increasingly used as molecular markers for population genetics and genotyping of different organisms. The technique exploits the hypervariability of DNA regions made of 10 to 20 or more tandem repeats of nucleotide couplets, triplets or quadruplets. Several polymorphic microsatellite loci have been identified in the genome of *C. albicans* and used in strain typing of the species (Table 1) (Bretagne et al. 1997; Metzgar et al. 1998; Botterel et al. 2001; Sampaio et al. 2003, 2005). The microsatellite loci *EF3* (Bretagne et al. 1997), *CDC3* and *HIS3* (Botterel et al. 2001) locate near coding regions, while the loci *ERK1, 2NF1, CCN2, CPH2* and *EFG1* (Metzgar et al. 1998) locate inside coding regions. More recently, several microsatellite loci called CAI, CAIII, CAV, CAVI and CAVII located in coding or noncoding regions have been employed for strain typing of *C. albicans* (Sampaio et al. 2003, 2005). The polymorphisms of microsatellite loci are usually detected using GeneScan or alike analysis with an automatic DNA sequencer and quantitatively designated by either the total lengths of the alleles (Bretagne et al. 1997; Botterel et al. 2001) or the total number of repeat units in the alleles (Sampaio et al. 2003, 2005). The quantitative designation has the advantage in interlaboratory data sharing and comparisons and database construction.

**Table 1.** Microsatellites in the genome of *C. albicans* that have been characterized for strain typing of the species.

Locus	Chromosome	Repetitive motif		Primer sequence (5′→3′)
CDC3	1	(AGTA)n	Fwd	CAGATGATTTTTTGTATGAGAAGAA
			Rev	CAGTCACAAGATTAAAATGTTCAAG
EF3	5	(TTTC)n(TTC)n	Fwd	TTTCCTCTTCCTTTCATATAGAA
			Rev	GGATTCACTAGCAGCAGACA
HIS3	2	(ATTT)n	Fwd	TGGCAAAAATGATATTCCAA
			Rev	TACACTATGCCCCAAACACA
ERK1	–	(CAGGCT)n(CAAGCT)n(CAA)n	Fwd	CGACCACGTCATCAATAGAAATCG
		(GCCGCA)n(CTT)n	Rev	CGTTGAATGAAACTTGACGAGGGG
CAI	4	(CAA)_2_CTG(CAA)n(CAG)n	Fwd	ATGCCATTGAGTGGAATTGG
			Rev	AGTGGCTTGTGTTGGGTTTT
CAIII	5	(GAA)n	Fwd	TTGGAATCACTTCACCAGGA
			Rev	TTTCCGTGGCATCAGTATCA
CAIV	–	(ATT)n	Fwd	TGCCAAATCTTGAGATACAAGTG
			Rev	CTTGCTTCTCTTGCTTTAAATTG
CAV	3	(ATT)n	Fwd	TGCCAAATCTTGAGATACAAGTG
			Rev	CTTGCTTCTCTTGCTTTAAATTG
CAVI	2	(TAAA)n	Fwd	ACAATTAAAGAAATGGATTTTAGTCAG
			Rev	TGCTGGTGCTGCTGGTATTA
CAVII	1	(CAAAT)n	Fwd	GGGGATAGAAATGGCATCAA
			Rev	TGTGAAACAATTCTCTCCTTGC

The discriminatory power for *C. albicans* strain typing with the microsatellites located near or inside coding regions was between 0.77 (for *CDC3*) and 0.91 (for *HIS3*). A higher value (0.97) was obtained when combining three loci (*CDC3, EF3* and *HIS3*) in a single multiplex amplification reaction (Botterel et al. 2001). Among the microsatellites used for *C. albicans* strain typing so far, the locus CAI located within the *RLM1* gene which codes for a transcription factor from the MADS box family (Sampaio et al. 2009) appeared to be the most polymorphic one, exhibiting a discriminatory power of 0.97 alone (Sampaio et al. 2003, 2005). Sequence analysis has revealed three different levels of polymorphism in microsatellites, (1) the total lengths or the number of repeats, (2) the structure of the repeated region and (3) point mutations outside the repeated region (Sampaio et al. 2003). In addition to the high cost, sequencing of microsatellites of *C. albicans* is a complicated procedure, because *C. albicans* is a diploid organism and the microsatellite loci are mostly heterozygous, thus cannot be sequenced directly. GeneScan technique which accurately determines DNA-fragment sizes is therefore commonly used in microsatellite analysis of *C. albicans.* The method can only detect the first level of microsatellite polymorphism, but the second and third levels of variation may contribute to further differentiation of *C. albicans* strains.

Li and Bai (2007) investigated an alternative approach to reveal the polymorphisms of microsatellites by utilizing the technique of single-strand conformation polymorphism (SSCP), a technique initially developed for point mutation detection in human DNA (Orita et al. 1989a, 1989b). Strain typing of a panel of 76 independent clinical isolates by PCR-SSCP analysis of CAI achieved a discriminatory power of 0.99 (Li & Bai 2007). Sequence comparison showed the advantage of SSCP over GeneSan analysis in the detection of point mutations in the microsatellite. The study demonstrated that PCR-SSCP analysis can reveal the three different levels of polymorphism in microsatellites simultaneously, thus being a powerful and economical approach for rapid strain typing of *C. albicans*.

## Multilocus sequence typing

Multilocus sequence typing (MLST) was initially developed for clone identification or strain typing of pathogenic bacteria (Maiden et al. 1998). The method analyses nucleotide polymorphisms of the sequences of approximately 500 bp internal fragments (loci) of housekeeping genes. For each locus, the different sequences present within a species are assigned as distinct alleles. The alleles at each of the sequenced loci define an allelic profile or sequence type of an isolate. Each isolate of a species can therefore be unambiguously characterized by a series of alleles at the housekeeping loci studied. The direct assignment of alleles based on nucleotide sequence polymorphisms of internal fragments from multiple housekeeping genes (usually six to eleven are required for bacteria) allows high levels of discrimination between isolates. A major advantage of MLST over other typing methods is that sequence data can be easily shared among laboratories in different countries and continents. Thus, permitting the establishment of one expanding online global database for each species concerned, and enabling exchange of molecular typing data via the Internet for global epidemiology (Maiden et al. 1998).

Before being formally called MLST, the method was used by mycologists to study basic evolutionary features of pathogenic fungi (Koufopanou et al. 1997; Xu et al. 2000; Anderson et al. 2001; Fisher et al. 2001). Bougnoux et al. (2002) examined the usefulness of MLST for characterization of clinical isolates of *C. albicans.* They sequenced the internal regions (loci) of six selected housekeeping genes of *C. albicans* and observed a wide variety of genotypes among the isolates studied. The high frequency of heterozygosity increased sequence diversity at each locus, thus allowed the identification of a greater number of genotypes or diploid sequence types (DSTs). The results showed that MLST is a highly discriminatory and reproducible method for unambiguous characterization of *C. albicans*. The method was then validated and optimized for *C. albicans* (Bougnoux et al. 2003; Tavanti et al. 2003), as a consequence, a set comprising the fragments of seven housekeeping genes *CaAAT1a, CaACC1, CaADP1, CaMPIb, CaSYA1, CaVPS13* and *CaZWF1b* is recommended for MLST with *C. albicans* (Table 2). A central internet database (http://calbicans.mlst.net) has been set up for deposition and analysis of *C. albicans* MLST data from any global source.

**Table 2.** A set of gene fragments recommended as an international standard for multilocus sequence typing of *C. albicans.*

Locus	Gene product	Chromosome		Primer sequence (5′→3′)	Amplicon sizes (bp)
AAT1a	Asparate aminotransferase	2	Fwd	ACTCAAGCTAGATTTTTGGC	478
			Rev	CAGCAACATGATTAGCCC	
ACC1	Acetyl-coenzyme A carboxylase	3	Fwd	GCAAGAGAAATTTTAATTCAATG	519
			Rev	TTCATCAACATCATCCAAGTG	
ADP1	ATP-dependent permease	1	Fwd	GAGCCAAGTATGAATGATTTG	537
			Rev	TTGATCAACAAACCCGATAAT	
MPIb	Mannose phosphate isomerase	2	Fwd	ACCAGAAATGGCCATTGC	486
			Rev	GCAGCCATGCATTCAATTAT	
SYA1	Alanyl-RNA synthetase	6	Fwd	AGAAGAATTGTTGCTGTTACTG	543
			Rev	GTTACCTTTACCACCAGCTTT	
VPS13	Vacuolar protein sorting protein	4	Fwd	TCGTTGAGAGATAATCGACTT	741
			Rev	ACGGATGGATCTCCAGTCC	
ZWF1b	Glucose-6-phosphate dehydrogenase	1	Fwd	GTTTCATTTGATCCTGAAGC	702
			Rev	GCCATTGATAAGTACCTGGAT	

Robles et al. (2004) assessed the value of MLST relative to those of other DNA-fingerprinting tools including RAPD, MLEE and RFLP with Ca3 probe hybridization techniques for discriminating among strains of *C. albicans*. The results demonstrated that the discriminatory power of MLST (99.6%) was higher than those of the other three methods compared. MLST analysis of *C. albicans* isolates recovered from the digestive tract of individuals within different families revealed intrafamilial transmission and microevolutions of the species. The data showed frequent colonization of a subject or several members of the same family by genetically indistinguishable or genetically close isolates. The genetically close isolates differed by loss-of-heterozygosity events at one or several of the MLST loci, suggesting the high frequency of microevolutions of commensal diploid *C. albicans* through the loss of heterozygosity (Bougnoux et al. 2006; Odds et al. 2006). MLST on a panel of 416 isolates of *C. albicans* from separate sources recognized a population structure comprising four major clades and eight minor clades (Tavanti et al. 2005). The relationship between clades of isolates and their properties of clinical relevance was showed from the data obtained. Different clades of *C. albicans* may differ significantly in the proportions of isolates from blood, the oropharynx, the vagina and other sites. When a larger panel of *C. albicans* isolates (1391) was subjected for MLST analysis, the number of clades recognized increased to 17 (Odds et al. 2007). ABC types (based on the presence or absence of an intron in rDNA, McCullough et al. 1999) and geographical origins showed statistically significant variations among clades, but anatomical source and antifungal-susceptibility data were not significantly associated. Computational haplotype analysis of the gene fragments sequenced for MLST showed a high frequency of recombination events, which suggests that *C. albicans* isolates had mixed evolutionary histories resembling those of a sexually reproducing species (Odds et al. 2007). In addition to nucleic housekeeping genes, mitochondrial genes have been demonstrated to be promising targets for genotyping and population genetics of *C. albicans* (Wang et al. 2007).

## Association of genotypes with infection types in *C. albicans*

Genotyping of *C. albicans* strains associated with different conditions of vulvovaginal candidosis (VVC) using PCR-SSCP analysis of CAI revealed for the first time that most *C. albicans* strains causing VVC possessed specific genotypes and that the genotype distribution of *C. albicans* strains correlated with the severity of VVC (Fan et al. 2008). The result provides a new clue and approach to elucidating the infection source of VVC. The genotype distributions of *C. albicans* strains associated with VVC of women and balanoposthitis of men and strains from various extragenital sites were investigated further by using GeneScan analysis of CAI (Li et al. 2008). The result showed that the CAI genotypes of independent *C. albicans* strains isolated from extragenital sites were mostly of individual specificity. In contrast, strains associated with VVC were mainly concentrated to a few genotypes, with CAI genotypes 30–45 and 32–46 being the most common. The distribution frequencies of *C. albicans* strains with the two dominant genotypes were significantly correlated with the severity of VVC. A similar genotype-distribution pattern of *C. albicans* strains associated with balanoposthitis was also revealed.

MLST analysis of 199 independent Chinese *C. albicans* with the focus on the isolates associated with VVC of Chinese women and 221 vaginal isolates from other countries available from the consensus MLST database of *C. albicans* showed that the majority of the VVC (71.6%) and balanitis (92.3%) isolates from China were located in clade 1 of *C. albicans;* while only 40.6% of the vaginal isolates and 7.8% of the oral isolates from healthy volunteers were found in the same clade (Ge et al. 2012). Furthermore, 69.1% of the VVC and 84.5% of the balanitis isolates concentrated in a cluster of clade 1 with DST 79 as the primary founder. The isolates in this cluster possessed microsatellite genotypes CAI 30–45, CAI 32–46 and their close derivatives. Interestingly, a remarkable difference in genotype-distribution patterns between Chinese and non-Chinese vaginal isolates of *C. albicans* was observed. Only 11.3% of the non-Chinese vaginal isolates compared were located in the cluster concentrated with Chinese VVC isolates (Ge et al. 2012). The results suggest (1) the existence of vaginopathic *C. albicans* strains with enhanced virulence and tropism for the vagina; (2) the high possibility of sexual transmission of genital *C. albicans* infections; and (3) the significant role of strain differences in the etiology of VVC. The significant association of specific genotypes with genital infections suggests that the identification of genotypes is of diagnostic and therapeutic significance.

MLST analysis also showed that particular *C. albicans* strains exist in the digestive tract of dyspeptic patients from China (Gong et al. 2012). Species-diversity investigation of the yeasts from the oral and gastric mucosa swab samples from patients who presented dyspeptic symptoms or ulcer complaints and those from oral swab samples from healthy volunteers showed that *C. albicans* was isolated from 97.8% of the *Candida*-positive subjects in the dyspeptic group, but from only 56.3% in the healthy group (*P* = 0.001). MLST analysis of the dominant species *C. albicans* showed that DST1593 was the dominant genotype from the digestive tract of the dyspeptic group (60%, 27/45), but not the healthy group (14.8%, 4/27) (*P* = 0.001). The result implies a possible link between particular *C. albicans* strain genotypes and host microenvironments and a positive detection of particular *C. albicans* genotypes could signify susceptibility to dyspepsia (Gong et al. 2012).

## Correlation between azole susceptibilities and genotypes in *C. albicans*

Antifungal-susceptibility testing using the Etest method revealed that the *C. albicans* isolates causing VVC in Chinese women were generally susceptible to fluconazole, amphotericin B, ketoconazole and flucytosine; however, 19.1% of the isolates could be interpreted as being resistant to itraconazole, *in vitro.* Interestingly, most of the itraconazole-resistant isolates belonged to a specific genotype as revealed by PCR-SSCP analysis of the CAI microsatellite (Liu et al. 2009). The relationship between susceptibilities to fluconazole and itraconazole and microsatellite CAI genotypes were further examined from a larger set of *C. albicans* isolates from the vagina and oral cavity of Chinese VVC patients and asymptomatic carriers (Ge et al. 2010). The CAI genotypes of these strains were identified using GeneScan analysis. Antifungal-susceptibility testing using the standard method (M27-A2) showed that the two dominant genotypes, CAI 30–45 and CAI 32–46 associated with vulvovaginitis showed significantly different azole-susceptibility patterns with strong statistical support. CAI 32–46 isolates were usually less susceptible to both fluconazole and itraconazole than CAI 30–45 isolates and than the oral isolates with other diversified CAI genotypes. Remarkably different mutation patterns in the azole target gene ERG11 were correspondingly observed among *C. albicans* isolates representing different genotypes and sources. Isolates with the same or similar CAI genotypes usually possessed identical or phylogenetically closely related *ERG11* sequences. Loss of heterozygosity in *ERG11* was observed in all the CAI 32–46 isolates but not in the CAI 30–45 isolates and most of the oral isolates sequenced. Compared with the *ERG11* sequence of strain SC5314, two homozygous missense mutations (G487T and T916C) leading to two amino-acid changes (A114S and Y257H) in the Erg11p were found in CAI 32–46 isolates (Ge et al. 2010). The correlation between azole-susceptibility and *C. albicans* genotype may be of potential therapeutic significance.

## Conclusion and perspective

Highly discriminative and portable molecular typing or DNA-fingerprinting methods have been developed for *C. albicans* in recent years and the application of these methods has resulted in interesting findings, including the association of genotypes with infection types; the possible link between particular strain types and host microenvironments and the correlation between azole susceptibilities and certain genotypes in *C. albicans*. It is interesting to note that the remarkable enrichment of certain MLST and CAI genotypes in the *C. albicans* population associated with urogenital infections has only been reported in China. The factors responsible for the extensive sharing of specific genotypes by the *C. albicans* isolates associated with VVC patients across China remain to be elucidated. Considering the factor that China is the only country in the world enforcing family-planning policy in recent decades, it will be interesting to investigate if the contraception or birth-control measures taken by Chinese women are responsible for the prevalence of the specific genotypes in vaginal isolates from China. The highly polymorphic microsatellite CAI is located within the *RLM1* gene which codes for a transcription factor from the MADS box family, which regulates the expression of genes involved in the cell wall integrity pathway in *Saccharomyces cerevisiae.* The number of the trinucleotide (CAA) repeats in the CAI locus determines the number of glutamine repeats in the C-terminal of *C. albicans* Rlm1p. A recent study showed that increased number of glutamine repeats in Rlm1p enhances the resistance of *C. albicans* to stress agents (Sampaio et al. 2009). Interestingly, the dominant *C. albicans* strains causing genital infections in China usually possess much longer CAI alleles than those from the oral cavity and other sources (Li et al. 2008; Ge et al. 2012). Elucidation of the function of Rlm1p in *C. albicans* will probably help to explain the enrichment of *C. albicans* strains with specific CAI genotypes in the vagina of VVC patients.

## Funding

The author's research on molecular typing and MLST of *C. albicans* was supported with [grant number 2013ZX10004612-003] from the Ministry of Science and Technology of China and [grant number 30825002] from the National Natural Science Foundation of China.
